# Monothiol and dithiol glutaredoxin-1 from *Clostridium oremlandii*: identification of domain-swapped structures by NMR, X-ray crystallography and HDX mass spectrometry

**DOI:** 10.1107/S2052252520011598

**Published:** 2020-09-19

**Authors:** Kitaik Lee, Kwon Joo Yeo, Sae Hae Choi, Eun Hye Lee, Bo Keun Kim, Sulhee Kim, Hae-Kap Cheong, Won-Kyu Lee, Hwa-Young Kim, Eunha Hwang, Ju Rang Woo, Sung-Joon Lee, Kwang Yeon Hwang

**Affiliations:** a Department of Biotechnology, School of Life Sciences and Biotechnology for BK21 PLUS, Institute of Life Science and Natural Resources, Korea University, 145 Anam-ro, Seongbuk-gu, Seoul 02841, Republic of Korea; b Structural Plant Biology Laboratory, Department of Botany and Plant Biology, University of Geneva, 1211 Geneva, Switzerland; c Division of Magnetic Resonance, Korea Basic Science Institute, 162 Yeongudanji-ro, Ochang, Chungbuk 28119, Republic of Korea; d New Drug Development Center, Osong Medical Innovation Foundation, Osong, Cheongju, Chungbuk 28160, Republic of Korea; e Department of Biochemistry and Molecular Biology, Yeungnam University College of Medicine, Daegu 38541, Republic of Korea

**Keywords:** domain swapping, oxidoreductases, disulfide bonds, glutaredoxin

## Abstract

Protein dimerization or oligomerization resulting from swapping part of the protein between neighboring polypeptide chains is known to play a key role in the regulation of protein function and in the formation of protein aggregates. Glutaredoxin-1 from *Clostridium oremlandii* (cGrx1) was used as a model to explore the formation of multiple domain-swapped conformations, which were made possible by modulating several hinge-loop residues that can form a pivot for domain swapping. Specifically, two alternative domain-swapped structures were generated and analyzed using nuclear magnetic resonance (NMR), X-ray crystallography, circular-dichroism spectroscopy and hydrogen/deuterium-exchange (HDX) mass spectrometry. The first domain-swapped structure (β3-swap) was formed by the hexameric cGrx1–cMsrA complex. The second domain-swapped structure (β1-swap) was formed by monothiol cGrx1 (C16S) alone. In summary, the first domain-swapped structure of an oxidoreductase in a hetero-oligomeric complex is presented. In particular, a single point mutation of a key cysteine residue to serine led to the formation of an intramolecular disulfide bond, as opposed to an intermolecular disulfide bond, and resulted in modulation of the underlying free-energy landscape of protein oligomerization.

## Abstract 2

Structures of monothiol and dithiol glutaredoxin-1 from *C. oremlandii* are reported. Monothiol glutaredoxin-1 formed domain-swapped structures, one of which was in a hetero-oligomer complex.

## 1. Introduction   

Three-dimensional (3D) domain swapping is a term for oligomerization in which two or more identical protein monomers exchange their structural domains (Bennett *et al.*, 1994 ▸). Since the introduction of the concept of domain swapping (Bennett *et al.*, 1994 ▸), many studies have established several structural elements that can control domain swapping, including proline or valine residues in the hinge loop of a protein (Bergdoll *et al.*, 1997 ▸, 1998 ▸; Rousseau *et al.*, 2001 ▸; Kuhlman *et al.*, 2001 ▸; Miller *et al.*, 2010 ▸; Shingate & Sowdhamini, 2012 ▸), the length of the hinge loop (Green *et al.*, 1995 ▸; Murray *et al.*, 1998 ▸; Picone *et al.*, 2005 ▸) and the formation of a disulfide bond (Yang *et al.*, 1999 ▸; Barrientos *et al.*, 2002 ▸, 2004 ▸; Knaus *et al.*, 2001 ▸). Among these elements, disulfide bonds provide a particularly useful approach to modulate domain swapping, as they can be formed intramolecularly like that in cyanovirin-N (Yang *et al.*, 1999 ▸; Barrientos *et al.*, 2002 ▸) and also intermolecularly as seen in the case of the human prion protein (Knaus *et al.*, 2001 ▸). Moreover, several studies have been performed to understand the energetic underpinning of domain swapping (Cho *et al.*, 2005 ▸; Yang *et al.*, 2004 ▸). Indeed, there is no reason why a protein should favor domain swapping; further, the domain-swapped configuration does not ensure that the protein is in its most stable configuration even though it is preferred at equilibrium (Cho *et al.*, 2005 ▸). However, the topology of the monomeric protein is sufficient to predict whether and determine how a protein will form domain-swapped complexes (Yang *et al.*, 2004 ▸). Several studies have outlined protein-specific methods for the design of domain-swapped complexes (Picone *et al.*, 2005 ▸; Orlikowska *et al.*, 2011 ▸; Kuhlman *et al.*, 2001 ▸; Reis *et al.*, 2014 ▸; Rousseau *et al.*, 2001 ▸; Pica *et al.*, 2013 ▸; Murray *et al.*, 1998 ▸); in some cases, structure-based models of protein folding have revealed the mechanism of domain swapping (Mascarenhas & Gosavi, 2016 ▸, 2017 ▸). Introducing intramolecular disulfide bonds could theor­etically affect the energy landscape and change the mechanism of folding for domain swapping (Cho *et al.*, 2005 ▸). Fusing a ‘lever’ protein into an internal position of a target protein that induces disulfide cross-linking can also lead to domain swapping (Ha *et al.*, 2012 ▸, 2015 ▸). In this study, we encountered inexplicable crystal structures of domain-swapped complexes during structural and mechanistic studies of glutaredoxin-1 from *Clostridium oremlandii* (strain OhILAs; cGrx1) and its complex with methionine sulfoxide reductase A from the same organism (cMsrA). Specifically, we determined the structures of both dithiol and monothiol versions of cGrx1 (d-cGrx1 and m-cGrx1, respectively) and the complex of m-cGrx1 with cMsrA, in which the structures of m-cGrx1 displayed two alternative domain-swapped configurations. Both cGrx1 and cMsrA are native seleno­proteins that contain a catalytic selenocysteine (Kim *et al.*, 2009 ▸), and cGrx1 is proposed to be a reductant of cMsrA (Kim *et al.*, 2015 ▸; Lee *et al.*, 2015 ▸). The thiol–disulfide oxido­reductase cGrx1 plays a role in maintaining the cellular redox homeostasis and contains a U(or C)P*X*C motif in its active site. In general, the first cysteine, *i.e.* the catalytic cysteine of the CP*X*C motif, attacks the cysteine residue of the target enzyme and is oxidized. The second cysteine, which is referred to as the resolving cysteine, then forms an intramolecular disulfide bond with the oxidized catalytic cysteine (Kim & Gladyshev, 2007 ▸; Boschi-Muller *et al.*, 2008 ▸; Lee *et al.*, 2015 ▸), which is subsequently reduced by a reductant, glutathione (GSH). Despite these relatively well established steps, mutation of the resolving cysteine leads to an intermolecular disulfide bond, causing domain swapping. The domain swapping was confirmed in solution by nuclear magnetic resonance (NMR) spectroscopy and hydrogen/deuterium-exchange (HDX) mass spectrometry (MS). Thus, through protein engineering, we can utilize the CP*X*C motif and its cysteine mutation to control protein oligomerization by inducing alternative domain swapping.

## 2. Experimental procedures   

### 2.1. Cloning, protein expression and purification   

cMsrA was expressed and purified as described previously (Kim *et al.*, 2009 ▸). Monothiol and dithiol cGrx1 were generated as described previously (Kim *et al.*, 2011 ▸). The catalytic selenocysteines of cMsrA and cGrx1 were replaced with cysteines. The column-purified proteins were concentrated to a final concentration of approximately 15 mg ml^−1^ as determined by the Bradford assay using bovine serum albumin as the standard. The m-cGrx1–cMsrA complex was purified using a Superdex 75 HiLoad 16/60 column (GE Healthcare) after incubation with 5 m*M* methionine sulfoxide at 4°C for 2 h. The column-purified protein complex was concentrated to a final concentration of approximately 20 mg ml^−1^.

### 2.2. Crystallization and data collection   

The sitting-drop vapor-diffusion method was used for initial crystallization screening at 20°C by applying various screening kits from Hampton Research (Crystal Screen, Index, SaltRx, PEG/Ion, PEGRx, Crystal Screen Cryo and Crystal Screen Lite) and Anatrace (MCSG Crystallization Suite MCSG-1–4). Optimization of the crystallization conditions was then carried out sequentially by the hanging-drop vapor-diffusion method using 24-well VDX plates (Hampton Research). Each drop was set up by mixing 1 µl concentrated protein solution with an equal volume of reservoir solution and was equilibrated against 500 µl reservoir solution. Crystals of d-cGrx1, domain-swapped m-cGrx1 and the cGrx1–cMsrA complex suitable for X-ray diffraction data collection were obtained using reservoir solutions consisting of 0.1 *M* sodium phosphate/citric acid pH 4.2, 0.2 *M* lithium sulfate, 20% PEG 1000, of 0.1 *M* HEPES pH 7.5, 20% PEG 400, 8% PEG 8000 and of 0.1 *M* sodium phosphate/citric acid pH 4.2, 0.4 *M* potassium phosphate (dibasic)/1.6 *M* sodium phosphate (monobasic), respectively.

### 2.3. Structure determination   

Each crystal was transferred to the reservoir solution containing 25% glycerol and then cooled in liquid nitrogen for cryoprotection. X-ray diffraction data were collected on beamline BL11C at Pohang Accelerator Laboratory. The wavelength of the synchrotron X-rays was 1.000 Å and the maximum high resolution was 2.8 Å. All diffraction images were collected using 1.0° oscillations with 1 s exposures from 0° to 360°, and were integrated and scaled using the *HKL*-2000 package (Otwinowski & Minor, 1997 ▸). The structures were determined by the molecular-replacement method using *Phaser* (McCoy *et al.*, 2007 ▸), and model building was performed by *AutoBuild* based on* SOLVE*/*RESOLVE* within the *Phenix* suite (Liebschner *et al.*, 2019 ▸). *Coot* (Emsley *et al.*, 2010 ▸) and the *phenix.refine* tool in *Phenix* were used for refinement. The statistics of structure refinement are provided in Table 1 ▸.

### 2.4. Heteronuclear single quantum correlation (HSQC) NMR experiments   

Hydrogen–deuterium exchange was performed using an HDX Manager (Waters, USA) equipped with a LEAP PAL autosampler (LEAP Technologies, USA). 40 µ*M* d-cGrx1 and m-cGrx1 were prepared in 10 m*M* potassium phosphate pH 7.0, and 5 m*M* TCEP was added for a reduced-condition sample. The samples were labeled with 15 volumes of deuterated buffer (10 m*M* potassium phosphate, D_2_O pD 7.0) at 20°C and incubated for various time points: 0.33, 10, 60 and 240 min. The exchange was quenched with an equal volume of a prechilled quench buffer (100 m*M* potassium phosphate, 0.1 *M* TCEP, 0.4 *M* guanidine–HCl pH 2.66 at 0°C). The protein was digested on an Enzymate immobilized pepsin column (Waters, USA) and the peptides were trapped on a pre-column (2.1 × 5 mm, ACQUITY BEH VanGuard) and separated using a C18 column (1 × 100 mm, ACQUITY BEH, 1.7 µm; Waters, USA) with a linear gradient of acetonitrile (5–95%) supplemented with 0.1% formic acid. Peptides were analyzed using a SYNAPT G2-Si mass spectrometer with IMS (Waters, USA). The peptic peptides were identified in undeuterated samples with *ProteinLynx Global SERVER* 3.0 (Waters, USA). To process the HDX-MS data, the amount of deuterium in each peptide was determined by measuring the centroid of the isotopic distribution using *DynamX* 3.0 (Waters, USA).

### 2.5. Thermal shift assay   

The d-cGrx1 and m-cGrx1 proteins were diluted to 0.5 mg ml^−1^ in buffer (20 m*M* HEPES pH 7.5, 100 m*M* NaCl) prior to loading. The samples were loaded and then heated from 25 to 85°C at 0.5°C min^−1^. The circular-dichroism absorbance at 222 nm was recorded using a circular-dichroism spectrophotometer (Jasco, Oklahoma City, Oklahoma, USA) and normalized to calculate the melting temperature of each protein.

## 3. Results and discussion   

**Figure 1:**
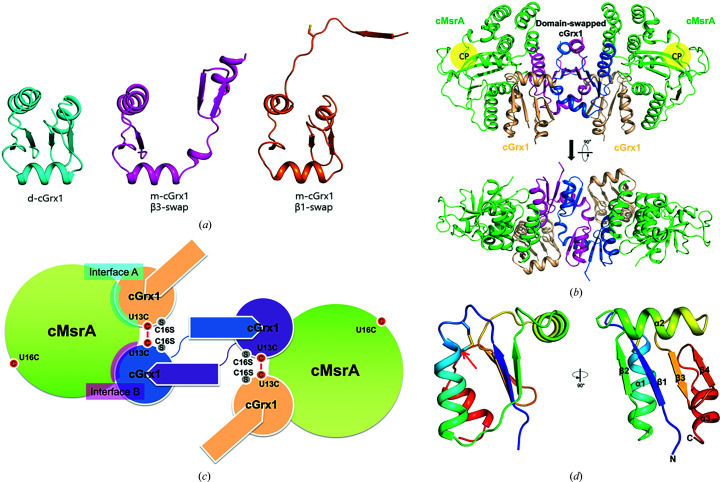
Structures of d-cGrx1, m-cGrx1 and the m-cGrx1–cMsrA complex. (*a*) d-cGrx1 is shown in cyan (left) and the domain-swapped structures of m-cGrx are shown in magenta (β3-swap; middle) and orange (β1-swap; right). (*b*) The heterohexameric state contains two cMsrA molecules and four cGrx1 molecules: cMsrA is in green, a domain-swapped dimer of cGrx1 is in blue and purple, and a disulfide dimer of cGrx1 is in beige. The catalytic pocket (CP) of cMsrA is indicated by a yellow circle. (*c*) Diagram of the heterohexamer of the monothiol cGrx1–cMsrA complex. The interfaces between cGrx1 and cMsrA are labeled in translucent boxes and disulfide bridges are shown as red lines. (*d*) The monomeric d-cGrx1 consists of three α-helices (α1, α2 and α3) and four β-sheets (β1, β2, β3 and β4). The N- and C-termini of d-cGrx1 are labeled N and C, respectively. Cys13 and Cys16 form a disulfide bond, which is represented as a stick.

**Figure 2:**
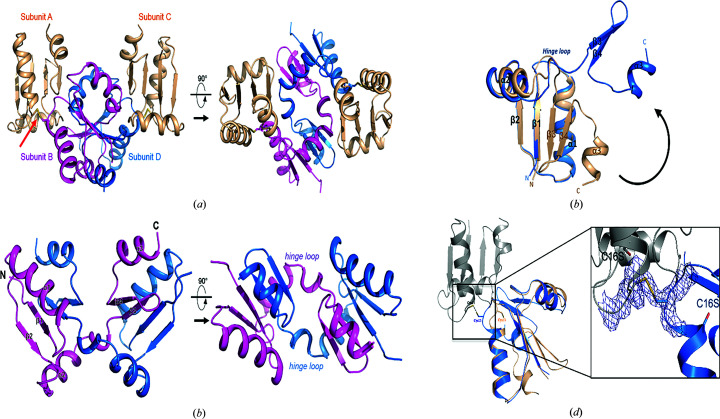
β3 domain-swapped structure of m-cGrx1. (*a*) Overall tetrameric structure of m-cGrx1. The disulfide dimers of m-cGrx1 are shown in beige (subunits *A* and *C*) and the domain-swapped dimers of m-cGrx1 are shown in purple and blue (subunits *B* and *D*). The red arrow indicates an intermolecular disulfide bridge between subunits *A* and *B*. (*b*) The domain-swapped m-cGrx1 monomers exchange β3, β4 and α3 with one another and are linked by a hinge loop. (*c*) The β3 domain-swapped m-cGrx1 structure (blue) reveals that the tertiary structure is unwound compared with that of d-cGrx1 (beige) and that the hinge loop is located between α2 and β3. (*d*) Comparison of the catalytic cysteine (Cys13) between domain-swapped m-cGrx1 and d-cGrx1. The superimposed domain-swapped m-cGrx1 (blue) and d-cGrx1 (beige) show that the catalytic cysteines (Cys13) are oriented differently. A disulfide dimeric m-cGrx1 is shown in gray. A 2*F*_o_ − *F*_c_ electron-density map is shown at the disulfide bridge. The domain-swapped m-cGrx1 (blue) and disulfide dimeric m-cGrx1 form a disulfide bridge. The 2*F*_o_ − *F*_c_ electron-density map is shown at 2.0σ.

We have determined three crystal structures: d-cGrx1 and domain-swapped structures (named β1-swap and β3-swap) of m-cGrx1 and the m-cGrx1–cMsrA complex [Fig. 1 ▸(*a*), Table 1 ▸]. The structure of d-cGrx1 revealed that an intramolecular disulfide bond was formed at the CPHC motif and that it exists as a dimer in the asymmetric unit. m-cGrx1, which was generated by mutating the resolving cysteine to serine (C16S), formed two different domain-swapped conformations, one in the presence of its substrate cMsrA (β3-swap) and the other in the presence of hydrogen peroxide (β1-swap). Similar to other glutaredoxins, cGrx1 is composed of three α-helices and four β-sheets, which together constitute the thioredoxin (Trx) fold (Martin, 1995 ▸) [Figs. 1 ▸(*a*) and 1 ▸(*d*), Table 1 ▸]. Our structure showed that the catalytic and resolving cysteine residues (Cys13 and Cys16, respectively) formed a disulfide bond. This is consistent with a previous study on cGrx2, which demonstrated that the conserved CPYC motif formed a disulfide bond under oxidizing conditions (Lee *et al.*, 2014 ▸). Previous studies on the catalytic mechanism of cGrx1 have suggested that it can directly reduce oxidized cMsrA in a 1:2 ratio (Kim *et al.*, 2015 ▸; Lee *et al.*, 2015 ▸). In our structure, cMsrA and cGrx1 formed a heterohexamer with an m-cGrx1 tetramer interposed with each cMsrA molecule [Figs. 1 ▸(*b*) and 1 ▸(*c*), Table 1 ▸]. The m-cGrx1 tetramer consisted of two disulfide dimers (subunits *AB* and *CD*), in which subunits *A* and *C* retained their normal tertiary structure, while the other two subunits (*i.e.* subunits *B* and *D*) formed a domain-swapped structure (Fig. 2 ▸). The β3 domain swapping, in particular, was formed by exchanging β3, β4 and α3 with each other. Surprisingly, the binding interface of cGrx1 was not close to the active-site cysteine of cMsrA, suggesting that this binding was unrelated to its activity unless a major structural change occurred prior to catalysis. Overall, our crystal structures revealed that cGrx1 forms different dimers characterized by different types of intermolecular interactions. d-cGrx1 formed a dimer by noncovalent interactions, including several hydrogen bonds. In contrast, the m-cGrx1 tetramer formed a dimer by disulfide bonding (Fig. 2 ▸). In the tetramer, the β3-swap structure suggested that domain swapping was made possible by unwinding of the hinge loop located between the α2 helix and the β3 sheet to allow the separation of β1 and β3 [Figs. 2 ▸(*b*) and 2 ▸(*c*)]. Nevertheless, the two domain-swapped m-cGrx1 molecules retained the same overall structure as the other two m-cGrx1 molecules, as well as that of d-cGrx1, except for the hinge loop and the catalytic cysteine (Cys13). The catalytic Cys13, in particular, had a significantly different orientation between d-cGrx1 and the β3-swap structure. Cys13 of d-cGrx1 formed a disulfide bond with its resolving cysteine, which pointed inwards, while Cys13 of the β3-swap structure was directed outwards to form a disulfide bond to another cGrx1 [Fig. 2 ▸(*d*)].

**Figure 3:**
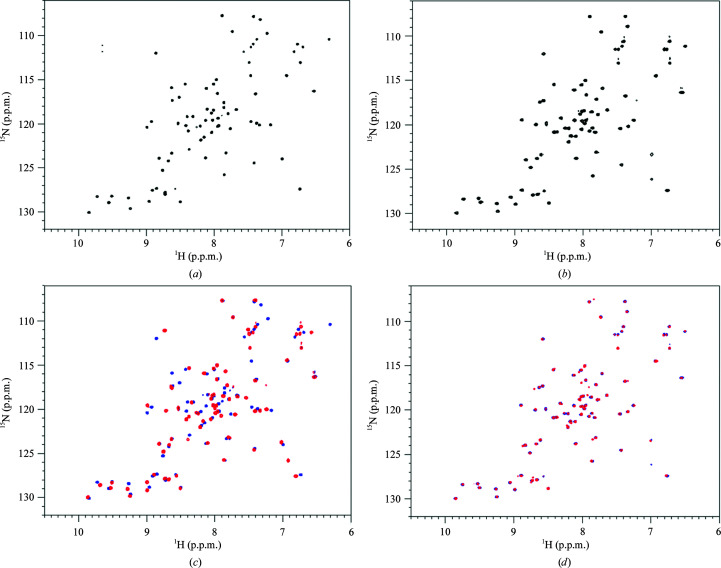
2D [^1^H,^15^N]-HSQC spectra of d-cGrx1 and m-cGrx1 recorded at 298 K on a Bruker Avance 800 MHz NMR spectrometer. (*a*) d-cGrx1. (*b*) m-cGrx1. (*c*, *d*) 2D [^1^H,^15^N]-HSQC spectra of cGrx1 with and without DTT. (*c*) d-cGrx1 in the absence of DTT is shown in blue and d-cGrx1 in the presence of DTT (5 m*M*) is shown in red.(*d*) m-cGrx1 in the absence of DTT is shown in blue and m-cGrx1 in the presence of DTT (5 m*M*) is shown in red.

To validate the observed domain swapping, we performed NMR spectroscopy. Based on our previous 3D NMR analysis of m-cGrx1 (Lee *et al.*, 2012 ▸), both d-cGrx1 and m-cGrx1 were labeled with ^15^N and we measured their 2D [^1^H,^15^N]-HSQC spectra (Fig. 3 ▸). While the HSQC spectra of ^15^N-m-cGrx1 were the same regardless of the presence of dithiothreitol (DTT), the corresponding spectra for ^15^N-d-cGrx1 exhibited a multiple peak shift in spectra with and without DTT (compare Figs. 3 ▸(*c*) and 3 ▸(*d*)]. This suggests that the intramolecular disulfide bond formed in d-cGrx1 induced a different conformation compared with the reduced cGrx1. Next, both ^15^N-labeled cGrx1s were incubated with cMsrA and methionine sulfoxide (Met-O) for 1 h at room temperature and the HSQC spectra were collected for each mixture. The overall peaks of ^15^N-d-cGrx1 were unchanged [Supplementary Fig. S1(*a*)]. On the other hand, the spectra of ^15^N-m-cGrx1 changed in a dose-dependent manner based on cMsrA/Met-O [Supplementary Fig. S1(*b*)]. More importantly, after the addition of DTT the HSQC spectra reverted to that of (reduced) m-cGrx1 alone [Supplementary Fig. S1(*c*)], suggesting that m-cGrx1 formed an oligomer or a complex with cMsrA in solution, triggered by the formation of the intermolecular disulfide bond. These results, as well as those for Grx1 alone, suggest that the conformation of (oxidized) m-cGrx1 complexed with cMsrA is different from those of d-cGrx1 and reduced m-cGrx1, supporting the domain-swapped conformation observed by X-ray crystallography (Supplementary Fig. S1).

**Figure 4:**
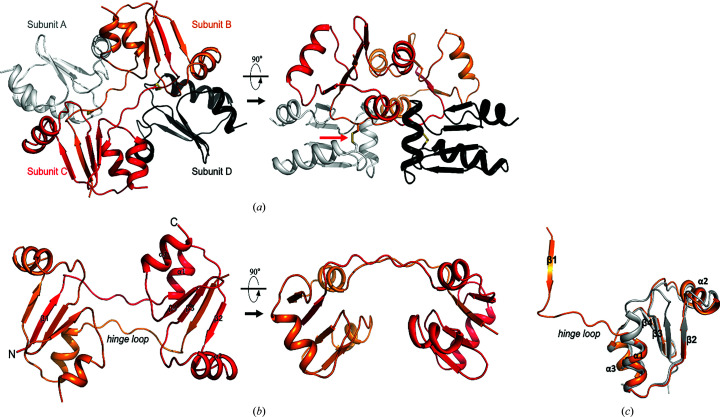
β1 domain-swapped structure of m-cGrx1. (*a*) The domain-swapped m-cGrx1 molecules (subunits *B* and *C*) are shown in red and orange and the disulfide dimeric m-cGrx1 molecules (subunits *A* and *D*) are shown in gray and black. The red arrow indicates the intermolecular disulfide bridge between subunits *A* and *C*. (*b*) The hinge loop is located between β1 and α1. (*c*) Structural comparison of d-cGrx1 and β1 domain-swapped m-cGrx1. The β1 domain-swapped m-cGrx1 (orange) reveals that the tertiary structure is unwound compared with that of d-cGrx1 (beige) and the hinge loop is located between α1 and β1. The α1 helix is partially unfolded.

In solution m-cGrx1 can exist in a multimeric form without cMsrA (Fig. 3 ▸) that may be the domain-swapped structure (β1-swap) in the crystal (Fig. 4 ▸, Table 1 ▸). The formation of the domain-swapped structure in solution was also supported by HSQC NMR spectroscopy (Supplementary Fig. S2). Like the β3-swap configuration, the β1-swap configuration formed a tetramer in which two monomers were domain swapped while the other two remained intact [Fig. 4 ▸(*a*)]. However, unlike the β3-swap structure, the β1-swap structure was formed by exchanging the β1 strands, which was made possible by partially unfolding the α1 helices in the respective domain-swapping partner [Figs. 4 ▸(*b*) and 4 ▸(*c*)]. Because of this domain swap, the monomers interacted with neighboring monomers through interfaces that differed from those formed in the β3-swap structure. Based on our structures, there are at least two domain-swapped configurations of m-cGrx1 that contain disulfide bonds. How can a single protein, *i.e.* m-cGrx1, adopt two alternative domain-swapped conformations? We expect that the answer pertains to the movement of the α1 helix. In the cMsrA–cGrx1 hexamer, m-cGrx1 interacts with cMsrA via two different interfaces (interfaces *A* and *B*, respectively), both of which involve the α1 helix [Fig. 1 ▸(*c*) and Supplementary Fig. S3(*a*)]. At these interfaces, Ser10, His15, Thr18, Lys20, Glu21 and Ser24 of the α1 helix participate in interactions with cMsrA. However, in the tetrameric m-cGrx1-only conformation, formation of an intact α1 helix was hampered by interactions with the extended loop residues from the domain-swapped partner [Supplementary Fig. S3(*b*)]. For example, Asn11, Thr12, Cys13 and His15 of the unfolded region of α1 interacted with His15, Phe66, Val53 and Met51 of the other monomer. Glu25 and Asn26 of α1 also formed hydrogen bonds to Lys43 and Lys46 of the α2 helix from the other monomer, respectively [Supplementary Fig. S3(*b*)]. Finally, the carbonyl O atom of His15 and the side chain of C16S engaged in a hydrogen-bond interaction between the domain-swapped monomers [Supplementary Fig. S3(*b*)].

**Figure 5:**
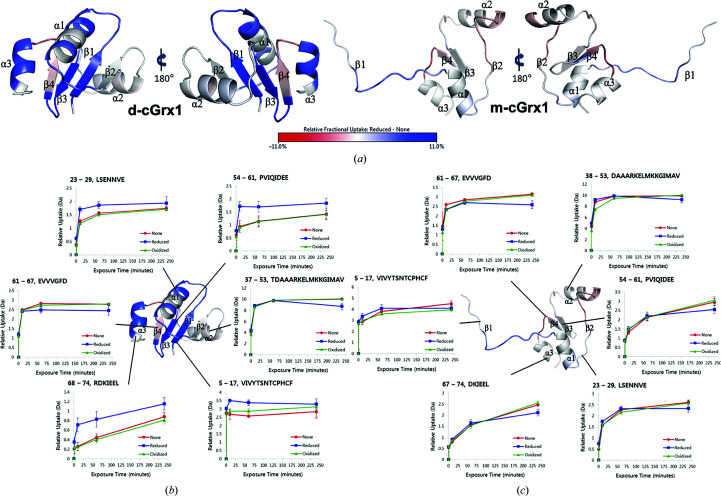
Deuterium uptake of d-cGrx1 and m-cGrx1. (*a*) The monomeric structures of d-cGrx1 and m-cGrx1 colored according to the differences in relative deuteration levels. Shades of red or blue reflect higher or lower deuterium uptake in the presence and absence of TCEP, respectively. (*b*, *c*) Uptake curves of selected peptides of d-cGrx1 and m-cGrx1. The observed relative deuterium uptake for each peptide, time point and condition were calculated and plotted against the labeling time. Error bars represent the average standard deviation observed across time points and replicates.

We carried out HDX experiments on d-cGrx1 and m-cGrx1 under reducing and oxidizing conditions. We obtained greater than 95% sequence coverage for both d-cGrx1 and m-cGrx1 in the HDX experiment, with a few exceptions (Fig. 5 ▸, Supplementary Figs. S4 and S5). HDX studies comparing d-cGrx1 and m-cGrx1 revealed that the deuterium uptake was significantly increased in m-cGrx compared with d-cGrx, especially in the β-sheet (β1, β3) and α-helical (α1, α3) regions (Fig. 5 ▸ and Supplementary Figs. S5). The increased deuterium levels in m-cGrx1 were owing to an open and solvent-accessible dynamic structure. Upon treatment with tris(2-carboxy­ethyl)phosphine (TCEP) to mimic the reduced form, the deuterium uptake was changed in the β-sheet (β1, β3) and α-helix (α1, α3) regions in d-cGrx but not in m-cGrx [Fig. 5 ▸(*c*) and Supplementary Figs. S5], indicating that that the reduction of the disulfide bond in the CPHC motif of d-cGrx1 loosens the tertiary structure. However, the deuterium uptake was decreased in m-cGrx1 after treatment with TCEP [Fig. 5 ▸(*c*) and Supplementary Fig. S5]. It is possible that disulfide dimerization of m-cGrx1 reduced its dynamic structure, including the domain swapping. Structural changes between β1 and β3 were frequently observed in both of the crystal structures of domain-swapped m-cGrx1, β3-swap and β1-swap, suggesting that the disulfide bond is crucial to the domain-swapped configuration of cGrx1 [Figs. 5 ▸(*b*) and 5 ▸(*c*)]. The deuterium uptake of the 5–17 peptide in m-cGrx1 in the oxidized form was lower than that in other conditions, suggesting that protein–protein interactions were modulated by domain swapping. The domain-swapped hexameric cMsrA–m-cGrx1 is the first reported structure with hetero-oligomeric domain swapping. To date, only two domain-swapped structures of thiol oxidoreductases have been reported, namely thioredoxin (Trx) from *Staphylococcus aureus* (Garcia-Pino *et al.*, 2009 ▸) and NrdH-redoxin from *Corynebacterium ammoniagenes* (Stehr & Lindqvist, 2004 ▸). However, from these structures it was not clear which factors, such as the mutation of specific residues, enable domain swapping. This is different in the case of m-cGrx1, where the domain swapping was triggered by the formation of the intermolecular disulfide bond and was not owing to the hinge loop interfering with the monomeric conformation of the protein. This was corroborated by the finding that conformational changes, including domain swapping, were only detected when the catalytic cysteine residue was oxidized. In addition, the domain-swapped configuration was absent in d-cGrx1. Together, these results suggest that the domain-swapped configuration of m-cGrx1 is independent of the hinge-loop sequence and is formed by altering the free-energy landscape of the whole protein owing to a change in disulfide bonding. A comparison of the thermostability of d-cGrx1 and m-cGrx1 also revealed a correlation between structural stability and the occurrence of intramolecular disulfide bonds (Supplementary Fig. S6). CD spectroscopy data for the thermal unfolding transition showed that d-cGrx1 was more stable than m-cGrx1 (Supplementary Table S1). The thermal unfolding of d-cGrx1 is characterized by an enthalpy change of 47.35 kcal mol^−1^ at the melting temperature (69.12°C), whereas for m-cGrx1 it is 50.34 kcal mol^−1^ at the melting temperature (53.42°C). In summary, using HDX we demonstrated that the intermolecular disulfide bond is crucial for domain swapping and maintenance of the tertiary structure of cGrx1. The domain swapping was validated in solution by 2D [^1^H,^15^N]-HSQC NMR spectroscopy. In conclusion, this study has shown that multiple domain-swapping conformations can be induced by a single mutation of m-cGrx1, allowing the formation of alternative intermolecular disulfide bridges. We believe that these findings provide a better understanding of the domain-swapping mechanism and act as novel examples of protein engineering.

## Supplementary Material

PDB reference: d-cGrx1, 7c10

PDB reference: m-cGrx1 (β1-swap), 7c12

PDB reference: m-cGrx1–cMsrA complex (β3-swap), 7c13

Supplementary Table and Figures. DOI: 10.1107/S2052252520011598/lz5041sup1.pdf

## Figures and Tables

**Table 1:** Data-collection and refinement statistics Values in parentheses are for the highest resolution shell.

	d-cGrx1	m-cGrx1–cMsrA complex (β3-swap)	m-cGrx1 (β1-swap)
Wavelength (Å)	1.0000	1.0000	1.0000
Resolution range (Å)	37.07–2.806 (2.907–2.806)	29.84–2.799 (2.899–2.799)	30.85–2.804 (2.904–2.804)
Space group	*C*121	*P*3_2_21	*P*3_1_
*a*, *b*, *c* (Å)	93.420, 61.105, 93.940	153.424, 153.424, 67.898	67.547, 67.547, 72.616
α, β, γ (°)	90, 104.237, 90	90, 90, 120	90, 90, 120
Total reflections	70031 (5110)	177562 (12154)	61774 (3729)
Unique reflections	12447 (1182)	22857 (2237)	9052 (891)
Multiplicity	5.6 (4.2)	7.8 (5.4)	6.8 (4.2)
Completeness (%)	97.81 (93.66)	99.69 (99.38)	99.49 (99.11)
Mean *I*/σ(*I*)	43.84 (15.55)	14.02 (3.52)	24.68 (4.34)
Wilson *B* factor (Å^2^)	55.61	36.96	51.16
*R*_merge_	0.04989 (0.1157)	0.1532 (0.4495)	0.09959 (0.402)
*R*_meas_	0.05437 (0.1299)	0.1635 (0.4969)	0.1069 (0.4587)
*R*_p.i.m._	0.02127 (0.0574)	0.05578 (0.2062)	0.03773 (0.215)
CC_1/2_	0.998 (0.987)	0.992 (0.229)	0.997 (0.264)
CC*	1.000 (0.997)	0.998 (0.61)	0.999 (0.646)
Reflections used in refinement	12443 (1181)	22857 (2237)	9050 (891)
Reflections used for *R*_free_	1245 (118)	2012 (200)	895 (92)
*R*_work_	0.2191 (0.2719)	0.2116 (0.3162)	0.2249 (0.2998)
*R*_free_	0.2753 (0.2955)	0.2454 (0.3428)	0.2793 (0.3595)
CC(work)	0.921 (0.857)	0.940 (0.473)	0.942 (0.476)
CC(free)	0.873 (0.753)	0.912 (0.443)	0.923 (0.324)
No. of non-H atoms	2918	2746	2312
No. of protein residues	369	344	292
R.m.s.d., bond lengths (Å)	0.003	0.003	0.005
R.m.s.d., angles (°)	0.56	0.61	1.09
Ramachandran favored (%)	97.5	93.2	93.3
Ramachandran allowed (%)	2.5	5.9	6.7
Ramachandran outliers (%)	0	0.89	0
Rotamer outliers (%)	0	0	0.4
Clashscore	3.94	6.26	9.09
Average *B* factor (Å^2^)	67.42	45.45	51.84

## References

[bb1] Barrientos, L. G., Lasala, F., Delgado, R., Sanchez, A. & Gronenborn, A. M. (2004). *Structure*, **12**, 1799–1807. 10.1016/j.str.2004.07.019 15458629

[bb2] Barrientos, L. G., Louis, J. M., Botos, I., Mori, T., Han, Z., O’Keefe, B. R., Boyd, M. R., Wlodawer, A. & Gronenborn, A. M. (2002). *Structure*, **10**, 673–686. 10.1016/s0969-2126(02)00758-x 12015150

[bb3] Bennett, M. J., Choe, S. & Eisenberg, D. (1994). *Proc. Natl Acad. Sci. USA*, **91**, 3127–3131. 10.1073/pnas.91.8.3127 PMC43528 8159715

[bb4] Bergdoll, M., Eltis, L. D., Cameron, A. D., Dumas, P. & Bolin, J. T. (1998). *Protein Sci.* **7**, 1661–1670. 10.1002/pro.5560070801 PMC2144073 10082363

[bb5] Bergdoll, M., Remy, M.-H., Cagnon, C., Masson, J.-M. & Dumas, P. (1997). *Structure*, **5**, 391–401. 10.1016/s0969-2126(97)00196-2 9083108

[bb6] Boschi-Muller, S., Gand, A. & Branlant, G. (2008). *Arch. Biochem. Biophys.* **474**, 266–273. 10.1016/j.abb.2008.02.007 18302927

[bb7] Cho, S. S., Levy, Y., Onuchic, J. N. & Wolynes, P. G. (2005). *Phys. Biol.* **2**, S44–S55. 10.1088/1478-3975/2/2/S05 16204848

[bb98] Emsley, P., Lohkamp, B., Scott, W. G. & Cowtan, K. (2010). *Acta Cryst.* D**66**, 486–501. 10.1107/S0907444910007493 PMC2852313 20383002

[bb8] Garcia-Pino, A., Martinez-Rodriguez, S., Wahni, K., Wyns, L., Loris, R. & Messens, J. (2009). *J. Mol. Biol.* **385**, 1590–1599. 10.1016/j.jmb.2008.11.040 19071139

[bb9] Green, S. M., Gittis, A. G., Meeker, A. K. & Lattman, E. E. (1995). *Nat. Struct. Mol. Biol.* **2**, 746–751. 10.1038/nsb0995-746 7552745

[bb10] Ha, J.-H., Karchin, J. M., Walker-Kopp, N., Castañeda, C. A. & Loh, S. N. (2015). *Chem. Biol.* **22**, 1384–1393. 10.1016/j.chembiol.2015.09.007 PMC4621486 26496687

[bb11] Ha, J.-H., Karchin, J. M., Walker-Kopp, N., Huang, L.-S., Berry, E. A. & Loh, S. N. (2012). *J. Mol. Biol.* **416**, 495–502. 10.1016/j.jmb.2011.12.050 PMC3288482 22245575

[bb12] Kim, H.-Y. & Gladyshev, V. N. (2007). *Biochem. J.* **407**, 321–329. 10.1042/BJ20070929 17922679

[bb13] Kim, H.-Y., Zhang, Y., Lee, B. C., Kim, J.-R. & Gladyshev, V. N. (2009). *Proteins*, **74**, 1008–1017. 10.1002/prot.22212 PMC2671064 18767149

[bb14] Kim, M.-J., Jeong, J., Jeong, J., Hwang, K. Y., Lee, K.-J. & Kim, H.-Y. (2015). *Biochem. Biophys. Res. Commun.* **457**, 567–571. 10.1016/j.bbrc.2015.01.025 25600814

[bb15] Kim, M.-J., Lee, B. C., Jeong, J., Lee, K.-J., Hwang, K. Y., Gladyshev, V. N. & Kim, H.-Y. (2011). *Mol. Microbiol.* **79**, 1194–1203. 10.1111/j.1365-2958.2010.07500.x PMC3062254 21210868

[bb16] Knaus, K. J., Morillas, M., Swietnicki, W., Malone, M., Surewicz, W. K. & Yee, V. C. (2001). *Nat. Struct. Biol.* **8**, 770–774. 10.1038/nsb0901-770 11524679

[bb17] Kuhlman, B., O’Neill, J. W., Kim, D. E., Zhang, K. Y. J. & Baker, D. (2001). *Proc. Natl Acad. Sci. USA*, **98**, 10687–10691. 10.1073/pnas.181354398 PMC58527 11526208

[bb18] Lee, E. H., Kim, E.-H., Kim, H.-Y., Hwang, K. Y. & Kim, H.-Y. (2012). *J. Anal. Sci. Technol.* **3**, 154–159.

[bb19] Lee, E. H., Kim, H.-Y. & Hwang, K. Y. (2014). *Arch. Biochem. Biophys.* **564**, 20–25. 10.1016/j.abb.2014.09.001 25218089

[bb20] Lee, E. H., Lee, K., Kwak, G.-H., Park, Y. S., Lee, K.-J., Hwang, K. Y. & Kim, H.-Y. (2015). *PLoS One*, **10**, e0131523. 10.1371/journal.pone.0131523 PMC4479559 26107511

[bb97] Liebschner, D., Afonine, P. V., Baker, M. L., Bunkóczi, G., Chen, V. B., Croll, T. I., Hintze, B., Hung, L.-W., Jain, S., McCoy, A. J., Moriarty, N. W., Oeffner, R. D., Poon, B. K., Prisant, M. G., Read, R. J., Richardson, J. S., Richardson, D. C., Sammito, M. D., Sobolev, O. V., Stockwell, D. H., Terwilliger, T. C., Urzhumtsev, A. G., Videau, L. L., Williams, C. J. & Adams, P. D. (2019). *Acta Cryst.* D**75**, 861–877. 10.1107/S2059798319011471 PMC6778852 31588918

[bb21] Martin, J. L. (1995). *Structure*, **3**, 245–250. 10.1016/s0969-2126(01)00154-x 7788290

[bb22] Mascarenhas, N. M. & Gosavi, S. (2016). *J. Phys. Chem. B*, **120**, 6929–6938. 10.1021/acs.jpcb.6b03968 27331242

[bb23] Mascarenhas, N. M. & Gosavi, S. (2017). *Prog. Biophys. Mol. Biol.* **128**, 113–120. 10.1016/j.pbiomolbio.2016.09.013 PMC7127520 27867057

[bb99] McCoy, A. J., Grosse-Kunstleve, R. W., Adams, P. D., Winn, M. D., Storoni, L. C. & Read, R. J. (2007). *J. Appl. Cryst.* **40**, 658–674. 10.1107/S0021889807021206 PMC2483472 19461840

[bb24] Miller, K. H., Karr, J. R. & Marqusee, S. (2010). *J. Mol. Biol.* **400**, 567–578. 10.1016/j.jmb.2010.05.017 PMC2911975 20471398

[bb25] Murray, A. J., Head, J. G., Barker, J. J. & Brady, R. L. (1998). *Nat. Struct. Mol. Biol.* **5**, 778–782. 10.1038/1816 9731771

[bb26] Orlikowska, M., Jankowska, E., Kołodziejczyk, R., Jaskólski, M. & Szymańska, A. (2011). *J. Struct. Biol.* **173**, 406–413. 10.1016/j.jsb.2010.11.009 21074623

[bb96] Otwinowski, Z. & Minor, W. (1997). *Methods Enzymol.* **276**, 307–326. 10.1016/S0076-6879(97)76066-X 27754618

[bb27] Pica, A., Merlino, A., Buell, A. K., Knowles, T. P. J., Pizzo, E., D’Alessio, G., Sica, F. & Mazzarella, L. (2013). *Acta Cryst.* D**69**, 2116–2123. 10.1107/S0907444913020507 24100329

[bb28] Picone, D., Di Fiore, A., Ercole, C., Franzese, M., Sica, F., Tomaselli, S. & Mazzarella, L. (2005). *J. Biol. Chem.* **280**, 13771–13778. 10.1074/jbc.M413157200 15647261

[bb29] Reis, J. M., Burns, D. C. & Woolley, G. A. (2014). *Biochemistry*, **53**, 5008–5016. 10.1021/bi500622x PMC4372075 25003701

[bb30] Rousseau, F., Schymkowitz, J. W., Wilkinson, H. R. & Itzhaki, L. S. (2001). *Proc. Natl Acad. Sci. USA*, **98**, 5596–5601. 10.1073/pnas.101542098 PMC33258 11344301

[bb31] Shingate, P. & Sowdhamini, R. (2012). *PLoS One*, **7**, e39305. 10.1371/journal.pone.0039305 PMC3407178 22848353

[bb32] Stehr, M. & Lindqvist, Y. (2004). *Proteins*, **55**, 613–619. 10.1002/prot.20126 15103625

[bb33] Yang, F., Bewley, C. A., Louis, J. M., Gustafson, K. R., Boyd, M. R., Gronenborn, A. M., Clore, G. M. & Wlodawer, A. (1999). *J. Mol. Biol.* **288**, 403–412. 10.1006/jmbi.1999.2693 10329150

[bb34] Yang, S., Cho, S. S., Levy, Y., Cheung, M. S., Levine, H., Wolynes, P. G. & Onuchic, J. N. (2004). *Proc. Natl Acad. Sci. USA*, **101**, 13786–13791. 10.1073/pnas.0403724101 PMC518834 15361578

