# Supplementary material for: Monothiol and dithiol glutaredoxin-1 from Clostridium oremlandii: identification of domain-swapped structures by NMR, X-ray crystallography and HDX mass spectrometry
Source: IUCrJ. 2020 Sep 19;7(Pt 6):1019–27. doi: 10.1107/S2052252520011598 (PMC7642778; doi:10.1107/S2052252520011598)
Supplement: Supplementary file 1 [file m-07-01019-sup1.pdf]

IUCrJ

Volume 7 (2020)

Supporting information for article:

Monothiol and dithiol glutaredoxin-1 from *Clostridium oremlandii*: identification of domain-swapped structures by NMR, X-ray crystallography and HDX mass spectrometry

Kitaik Lee, Kwon Joo Yeo, Sae Hae Choi, Eun Hye Lee, Bo Keun Kim, Sulhee Kim, Hae-Kap Cheong, Won-Kyu Lee, Hwa-Young Kim, Eunha Hwang, Ju Rang Woo, Sung-Joon Lee and Kwang Yeon Hwang

Table S1 Melting temperature of d-cGrx1 and m-cGrx1 based on the CD absorbance at 222nm.

	Tm (℃)	Tm error (＋/－)	ΔH (kcal/mol)
Dithiol cGrx1	69.12	0.055	47.35
Monothiol cGrx1	53.42	0.102	50.34

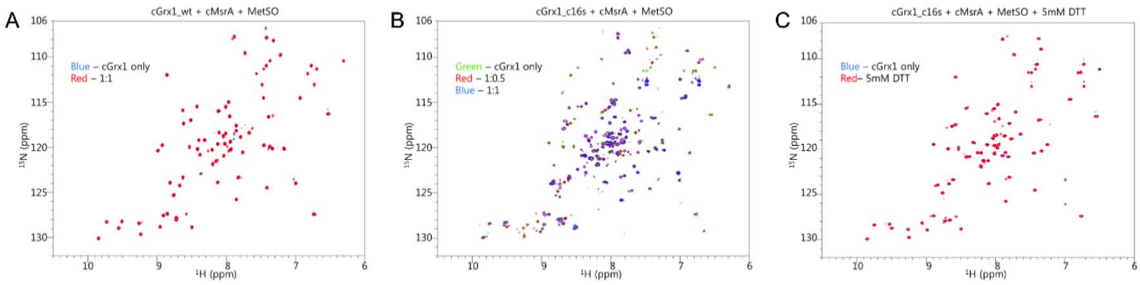

Figure S1 Comparison of 2D [^{1}H, ^{15}N]-HSQC spectra. (*a*) d-cGrx1 with cMsrA and 5mM Met-O. d-cGrx1 in alone is shown as *blue* and in presence of cMsrA and 5mM Met-O is shown as *red* peaks. (*b*) m-cGrx1 with cMsrA and 5mM Met-O. m-cGrx1 in alone is shown as *green*, in molar ratio of 1:0.5 with cMsrA is shown as *red* and in molar ratio of 1:1 with cMsrA is shown as *blue* peaks. (*c*) m-cGrx1 with 5mM DTT after incubating with cMsrA and 5mM Met-O. m-cGrx1 in alone is shown as *blue*, and with 5mM DTT after incubating with cMsrA and 5mM Met-O is shown as *red* peaks.

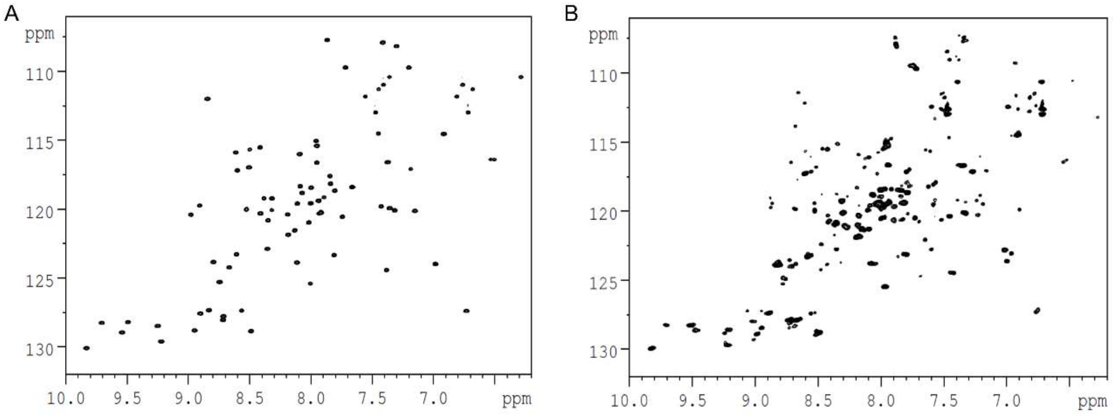

Figure S2 2D [^{1}H, ^{15}N]-HSQC spectra of m-cGrx1 and d-cGrx1 with 5mM oxidized Glutathione (GSSG). (*a*) 2D [^{1}H, ^{15}N]-HSQC spectra of d-cGrx1 with 5mM GSSG. (*b*) 2D [^{1}H, ^{15}N]-HSQC spectra of m-cGrx1 with 5mM GSSG.

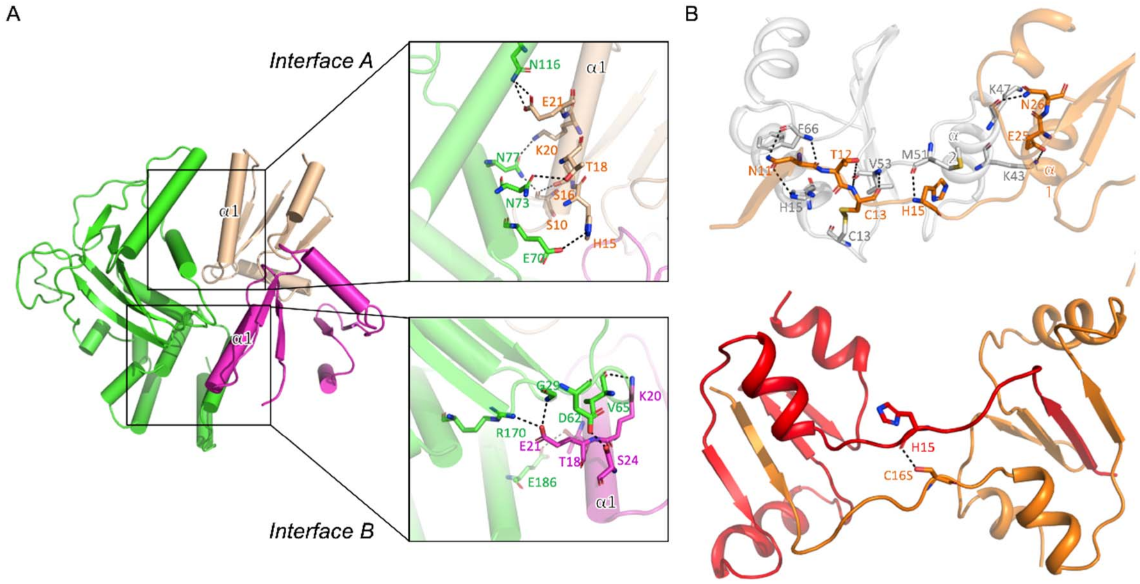

Figure S3 (*a*) Close view of interfaces between m-cGrx1 and cMsrA. *Interface A* and *Interface B* indicate the interface between disulfide dimeric m-cGrx1 and cMsrA, domain-swapping m-cGrx1 and cMsrA, respectively. The hydrogen bonds are shown as dash line. (*b*) Interactions of subunits of β1-swap. Interactions between disulfide dimeric m-cGrx1 (grey) and hinge loop of domain-swapping m-cGrx1 (orange). Interaction between each hinge loop of domain-swapped dimer. The hydrogen bonds are shown as dash line.

d-cGrx1

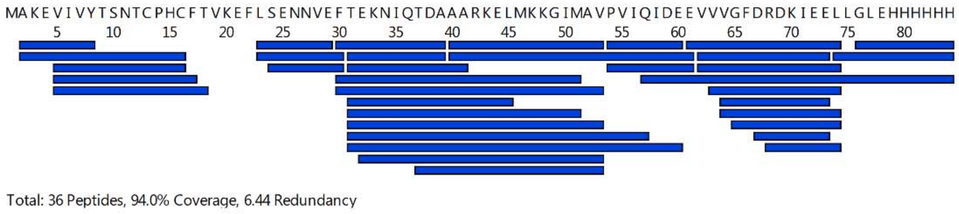

m-cGrx1(C16S)

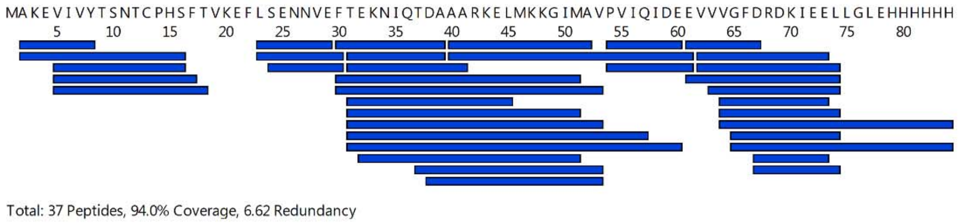

Figure S4 Sequence coverage of cGrx1. The peptides of cGrx1 obtained via peptide digestion and LC-IMS/MS analysis was shown as blue bars along the sequence.

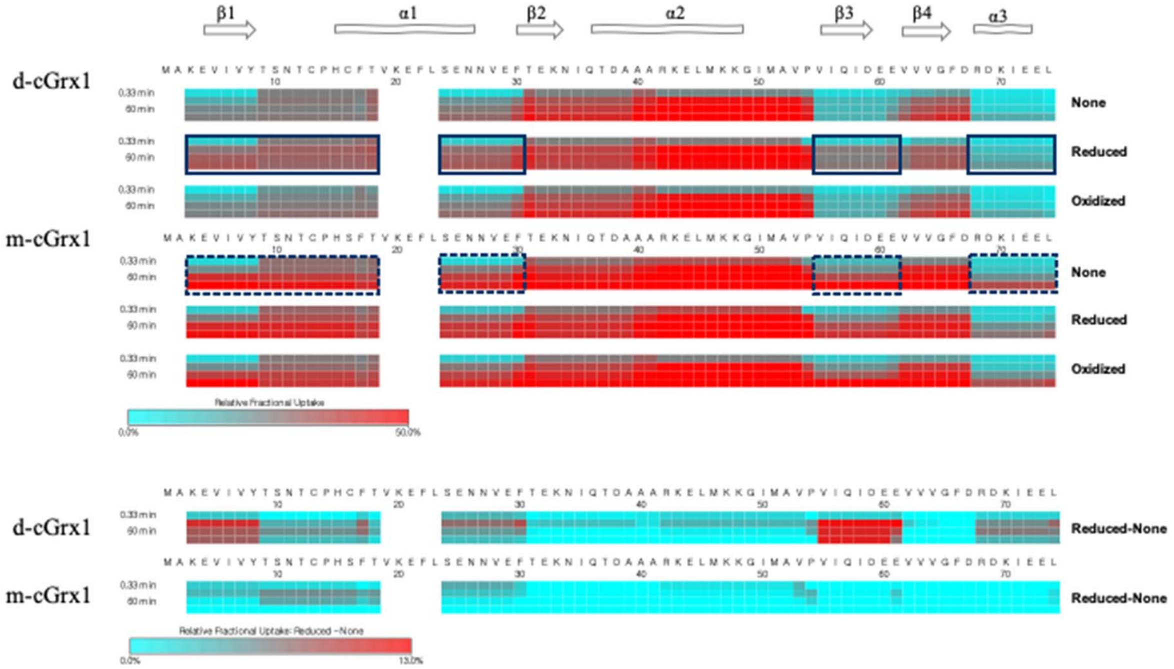

Figure S5 Heat maps of differential deuterium uptake for d- and m-cGrx1. The differential deuterium uptake, observed in the presence of TCEP of d-cGrx, is shown as bold box. The differential deuterium uptake between d-cGrx and m-cGrx1 is shown as dashed box.

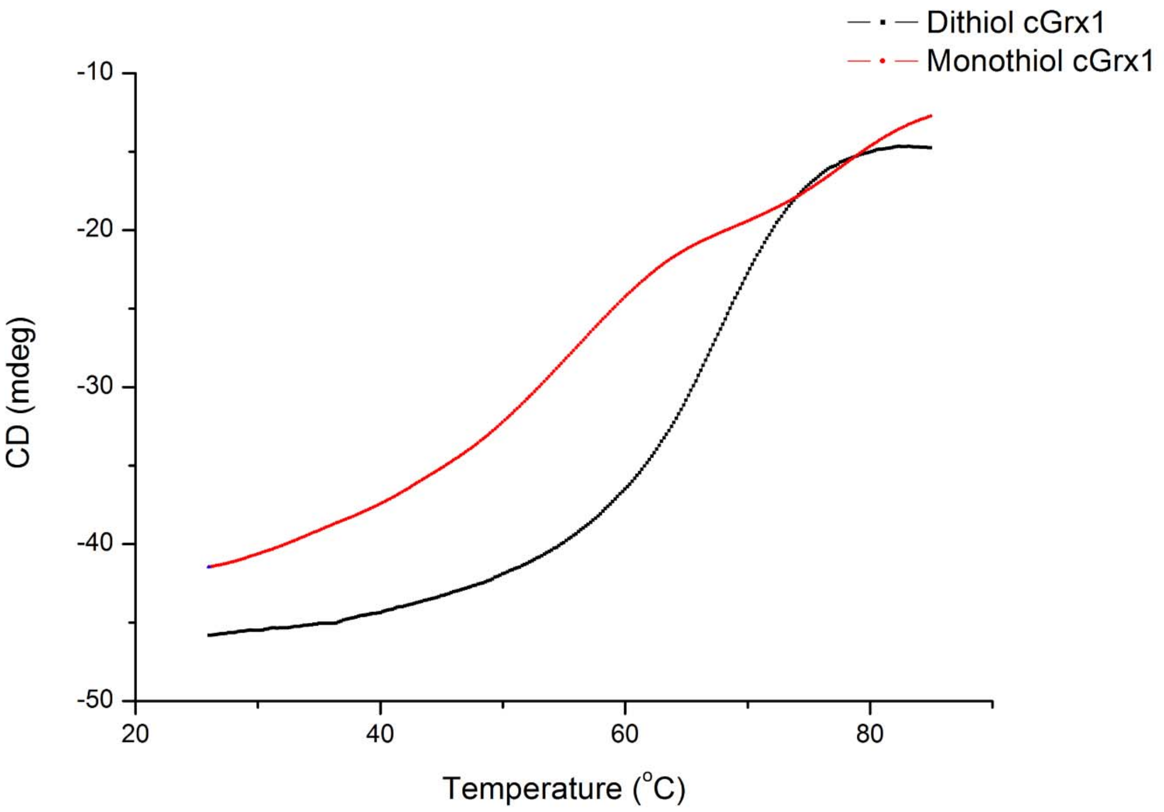

Figure S6 Thermal stability of cGrx1s monitored by CD spectroscopy. Thermal stability of d-cGrx1 (black) and m-cGrx1(red).
